# Retraction Note: Protective role of arachidonic acid against diabetic myocardial ischemic injury: a translational study of pigs, rats, and humans

**DOI:** 10.1186/s12933-025-02822-5

**Published:** 2025-06-23

**Authors:** Yunhui Lv, Kai Li, Shuo Wang, Xiaokang Wang, Guangxin Yue, Yangyang Zhang, Xin Lv, Ping Zhao, Shiping Wang, Qi Zhang, Qiuju Li, Jinyan Zhu, Jubo Li, Peng Peng, Yue Li, Jiafei Luo, Xue Zhang, Jianzhong Yang, Baojie Zhang, Xuemin Wang, Min Zhang, Chen Shen, Xin Wang, Miao Wang, Zhen Ye, Yongchun Cui

**Affiliations:** 1 Fuwai Hospital, State Key Laboratory of Cardiovascular Disease, National Center for Cardiovascular Diseases Beijing Key Laboratory of Pre-Clinical Research and Evaluation for Cardiovascular Implant Materials, Chinese Academy of Medical Sciences and Peking Union Medical College, 167 Beilishi Road, Xicheng District, Beijing, 100037 China; 2 Department of Pharmacy & Cardiology & Endocrinology & General Surgery, Suqian First Hospital, 120 Suzhi Road, Sucheng District, Suqian, 223800 Jiangsu China; 3 Department of Cardiothoracic Surgery, Nanjing Drum Tower Hospital, The Affiliated Hospital of Nanjing University Medical School, Nanjing, 210008 China

**Retraction Note to: Cardiovascular Diabetology (2024) 23:58** 10.1186/s12933-024-02123-3

The Editor-in-Chief has retracted this article because of concerns regarding the figures presented in this work. An investigation conducted after its publication identified the following issues:

- A portion of the panel in the second column and second row of Fig. 3G appears to overlap, when rotated and re-scaled, with a portion of the panel in the second column and fourth row in the same figure;
- Portions of the panel in the second column and first row of Fig. 4A appear to overlap with portions of the panel in the second column and second row in the same figure;
- Portions of the panel in the first column and first row of Fig. 4C appear to overlap with portions of the panel in the third column and second row in the same figure;
- Portions of the panel in the fourth column and second row of Fig. 4E appear to overlap, when rotated and re-scaled, with portions of the panel in the fourth column and sixth row in the same figure;
- A portion of the panel in the second column and second row of Fig. 7C appears to overlap, when re-scaled, with a portion of the panel in the fourth column and second row in the same figure;
- Multiple panels showing TUNEL assay results in Fig. 7C appear to overlap with panels in Fig. 6I in [1].

The panels in question represent cells or tissues subject to different experimental conditions. The Editor-in-Chief therefore no longer has confidence in the integrity of the research presented in this article.

Yongchun Cui, Yunhui Lv, Shiping Wang, Miao Wang, Xuemin Wang, Shuo Wang, Guangxin Yue, Qi Zhang, Yangyang Zhang, and Xue Zhang agree with this retraction. The remaining authors did not reply to correspondence from the Publisher about this retraction.
